# *Clitoria ternatea* Flower Petal Extract Inhibits Adipogenesis and Lipid Accumulation in 3T3-L1 Preadipocytes by Downregulating Adipogenic Gene Expression

**DOI:** 10.3390/molecules24101894

**Published:** 2019-05-17

**Authors:** Poramin Chayaratanasin, Allen Caobi, Chaturong Suparpprom, Sudarat Saenset, Porntip Pasukamonset, Nipattra Suanpairintr, Manuel Alejandro Barbieri, Sirichai Adisakwattana

**Affiliations:** 1 Department of Pharmacology, Faculty of Veterinary Sciences, Chulalongkorn University, Bangkok 10330, Thailand; jayporamin@gmail.com (P.C.); nipattra@gmail.com (N.S.); 2 Program in Veterinary Biosciences, Faculty of Veterinary Sciences, Chulalongkorn University, Bangkok 10330, Thailand; 3 Department of Biological sciences, Florida International University, Miami, FL 33199, USA; acaob001@fiu.edu (A.C.); barbieri@fiu.edu (M.A.B.); 4 Department of Chemistry and Center of Excellence for Innovation in Chemistry, Faculty of Science, Naresuan University, Ta-po, Phitsanulok 65000, Thailand; chaturongs@nu.ac.th (C.S.); saenset.s@gmail.com (S.S.); 5 Department of Home Economics, Faculty of Agriculture, Kasetsart University, Bangkok 10900, Thailand; pasukamonset@gmail.com; 6 Department of Nutrition and Dietetics, Faculty of Allied Health Sciences, Chulalongkorn University, Bangkok 10330, Thailand

**Keywords:** *Clitoria ternatea* extract, adipogenesis, lipolysis, inhibition, adipocytes

## Abstract

*Clitoria ternatea* (commonly known as blue pea) flower petal extract (CTE) is used as a natural colorant in a variety of foods and beverages. The objective of study was to determine the inhibitory effect of CTE on adipogenesis in 3T3-L1 preadipocytes. The phytochemical profiles of CTE were analyzed by liquid chromatography and tandem mass spectrometry (LC-MS/MS). Anti-adipogenesis effect of CTE was measured by using Oil Red O staining, intracellular triglyceride assay, quantitative real-time PCR and western blot analysis in 3T3-L1 adipocytes. Cell cycle studies were performed by flow cytometry. Lipolysis experiments were performed using a colorimetric assay kit. In early stages, CTE demonstrated anti-adipogenic effects through inhibition of proliferation and cell cycle retardation by suppressing expression of phospho-Akt and phospho-ERK1/2 signaling pathway. The results also showed that CTE inhibited the late stage of differentiation through diminishing expression of adipogenic transcription factors including PPARγ and C/EBPα. The inhibitory action was subsequently attenuated in downregulation of fatty acid synthase and acetyl-CoA carboxylase, causing the reduction of TG accumulation. In addition, CTE also enhanced catecholamine-induced lipolysis in adipocytes. These results suggest that CTE effectively attenuates adipogenesis by controlling cell cycle progression and downregulating adipogenic gene expression.

## 1. Introduction

Obesity is one of leading factors in the metabolic syndrome caused by an imbalance between food intake and energy expenditure [1,2]. Nowadays, obesity is a growing global health problem, responsible for the development of type 2 diabetes, cardiovascular diseases and atherosclerosis [3,4,5]. Obesity is mainly associated with increased expansion of white adipose tissue through the activation of adipogenesis [5]. The process of adipogenesis involves the changes of cell morphology from fibroblast-like shape of preadipocytes to mature and increased lipid synthesis and accumulation in adipocytes. Adipogenesis is generally described as a two-step process including cell proliferation and differentiation, resulting in an increase of the number (hyperplasia) and size (hypertrophy) of adipocytes. In early stage, pre-adipocytes are proliferated through activation of Akt and ERK signaling pathway. After the proliferative phase, they undergo to the formation of mature adipocytes from fibroblast-like pre-adipocyte into spherical shape. Accumulation of the triglyceride (TG) in mature adipocyte is activated by expression of adipogenic transcriptional factors including PPARγ and C/EBPα leading to regulation of fatty acid synthase (FAS) and acetyl-CoA carboxylase (ACC) [6]. Moreover, Akt1 and MAPK signaling pathway plays a pivotal role in regulating adipogenesis from cell proliferation to differentiation [7,8,9]. Activation of Akt1 contributes to promote cell cycle progression and terminal differentiation into mature adipocyte [10]. It has shown that activation of Akt1 markedly inhibited p21 and p27 (Cdk inhibitors) and subsequently triggered Cdk2 in the cell cycle progression [10,11]. As a result, mature adipocytes expand in size through the increased storage of triglycerides [10]. Besides, these can be hydrolyzed by hormone sensitive lipase (HSL) under deprivation status for energy homeostasis in response to catecholamines and insulin [12]. Apart from the Akt pathway, the ERK signaling pathway also contributes to the commitment effect in adipogenesis by initiating the proliferative step and increasing differentiation in adipocytes [13,14].

To date, there has been considerable interest in studying the effect of dietary plants on the prevention of obesity because they are largely free from side effects [15,16,17]. *Clitoria ternatea* L. (family: Fabaceae) or butterfly pea is widely distributed in tropical countries, South and Central America. This plant has been used for centuries in foods and medicines. In Asia and America, it is traditionally recommended for the treatment of snakebites, scorpion stings, chronic bronchitis, indigestion, constipation, fever, arthritis, eye ailments, sore throats, skin diseases, rheumatism, syphilis, eye and ear-diseases [18]. The flower petals of *C. ternatea*, which contain abundant anthocyanin pigments have considerable potential for application as a source of natural colorants in a variety of foods and beverages. The phytochemical components of *C. ternatea* flower extract (CTE) are mainly delphinidin-derived anthocyanins, including delphinidin-3,5-glucoside, delphinidin-3-glucoside and six major delphinidin-based ternatins (ternatins A1, A2, B1, B2, D1 and D2) [19,20]. CTE has been reported to possess various pharmacological effects such as antioxidant, antiglycation, antimicrobial, anti-platelet aggregation, anti-inflammatory, antipyretic, and antihelmintic activity [18,21,22]. In addition, CTE exerts antihyperglycemic and antihyperlipidemic effects in alloxan-induced diabetic rats [23,24]. Recently, CTE has shown anti-glycation activity through inhibition of formation of AGEs in fructose-glycated bovine serum albumin (BSA) [21]. It also prevents oxidative damage to BSA due to its free radical scavenging ability [21]. Although the antidiabetic and antiglycation activity of CTE has been well documented, studies regarding the effect of CTE on adipogenesis remain unknown. Therefore, the objective of current study was to elucidate the inhibitory effect of CTE and its underlying cellular mechanisms on adipogenesis and lipid accumulation by using 3T3-L1 cells.

## 2. Results

### 2.1. Characterization and Identification of Phenolic Compounds in CTE by LC-MS/MS

**Figure 1:**
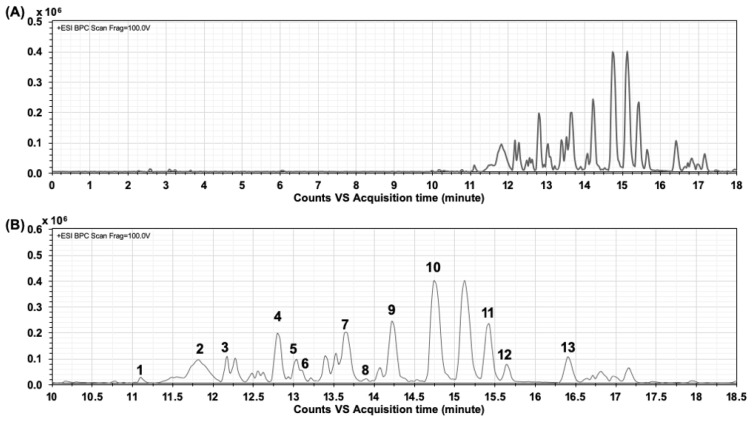
LC-MS/MS chromatogram of the *Clitoria ternatea* flower extract (CTE). (**A**) The expanded range of CTE chromatogram during 0–18 min. (**B**) The profile of CTE chromatogram (the number indicates isolated compounds).

**Figure 2:**
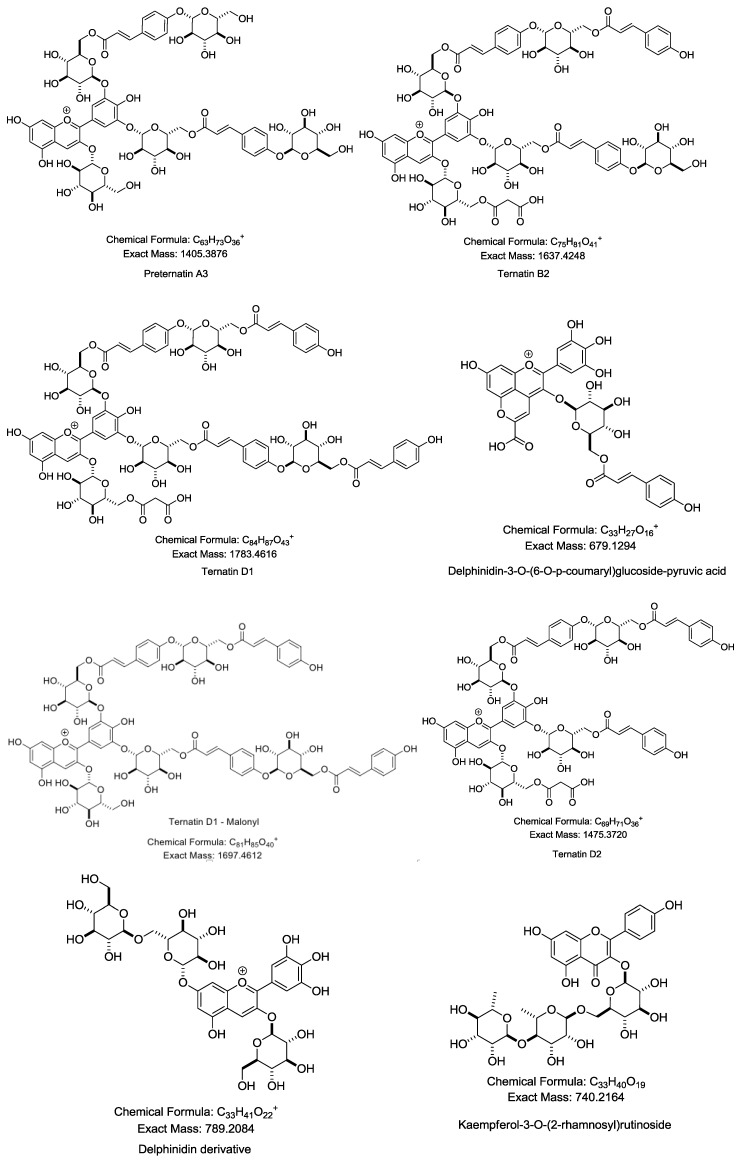

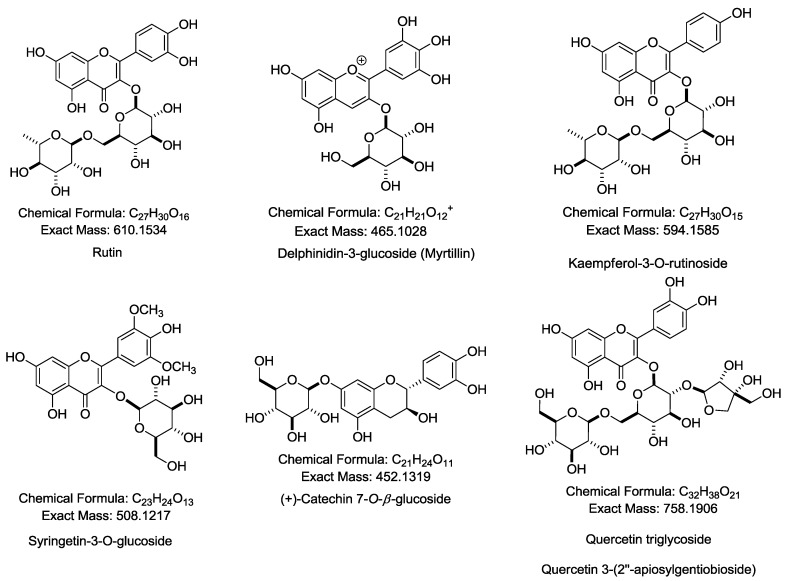
Structures of identified compounds listed in Table 1.

From the chromatograms obtained by LC/MS/MS (Figure 1), 14 compounds were identified based on their retention times, high-resolution mass spectral data (MS and MS/MS) of the fragment ions, and comparisons with previously published literature [25,26]. As reported in Table 1, the compounds were identified as preternatin A3, ternatin B2, ternatin D2, quercetin-3-rutinoside, ternatin D1, kaemferol-3-*O*-(2-rhamnosyl) rutinoside, delphinidin-3-glucoside, kaemferol-3-*O*-rutinoside, delphinidin-3-*O*-(6-*O*-*p*-coumaryl)glucoside-pyruvic acid, (+)-catechin 7-*O*-β-glucoside, syringetin-3-*O*-glucoside, quercetin triglycoside, and delphinidin derivatives (Figure 2).

### 2.2. Effects of CTE on Cell Viability of 3T3-L1 Cells

**Figure 3:**
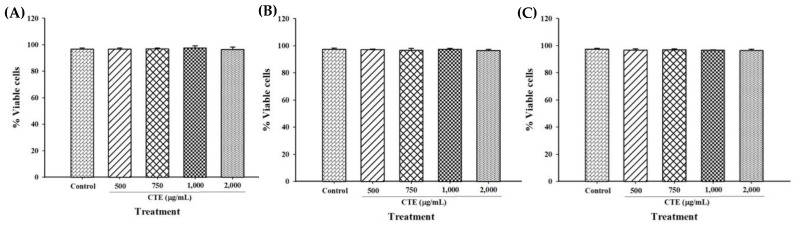
Effects of CTE on cell viability of 3T3-L1 cells by trypan blue assay at day 1 (**A**), day 3 (**B**) and day 9 (**C**). Each value represents the mean ± SEM (*n* = 3).

To determine whether CTE has cytotoxicity to 3T3-L1 cells, various concentrations of CTE (500–2000 µg/mL) were incubated with 3T3-L1 cells during adipogenesis process. There was no toxicity of CTE to 3T3-L1 cells at the concentration up to 2000 µg/mL at day 1, 3 and 9 (before and after differentiation) (Figure 3A–C).

### 2.3. Effects of CTE on the Cell Cycle of 3T3-L1 Cells

**Figure 4:**
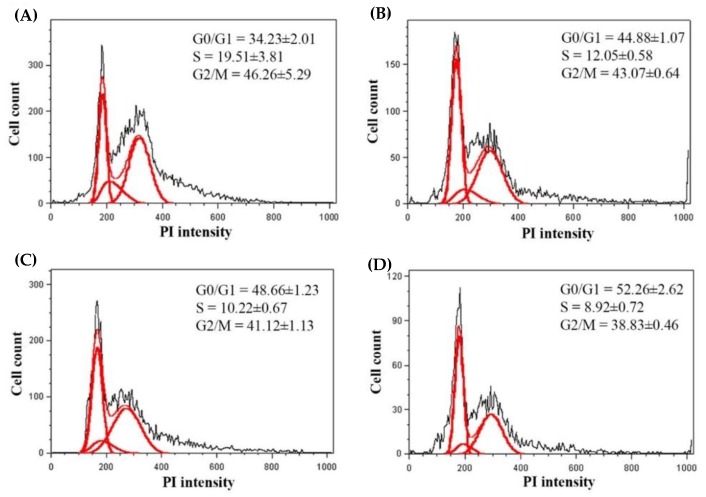
Effects of CTE on cell cycle of 3T3-L1 cells at day 1. Histograms are shown as the control group (**A**), CTE 250 µg/mL (**B**), 500 µg/mL (**C**), 750 µg/mL (**D**). Each value represents the mean ± SEM (*n* = 3).

As shown in Figure 4A–D, the results showed that CTE at concentration of 250, 500 and 750 µg/mL significantly delayed the cell cycle by increasing the cell distribution in G0/G1 phase from control at 34.23 ± 2.01% to 44.88 ± 1.07%, 48.66 ± 1.23% and 52.26 ± 2.62%, respectively. In addition, CTE concentration-dependently decreased of G2/M phase from control at 46.26 ± 5.29 to 43.07 ± 0.64, 41.12 ± 1.13 and 38.83 ± 0.46%, respectively.

### 2.4. Effects of CTE on Akt1 and ERK1/2 Signaling Pathways in 3T3-L1 Cells

**Figure 5:**
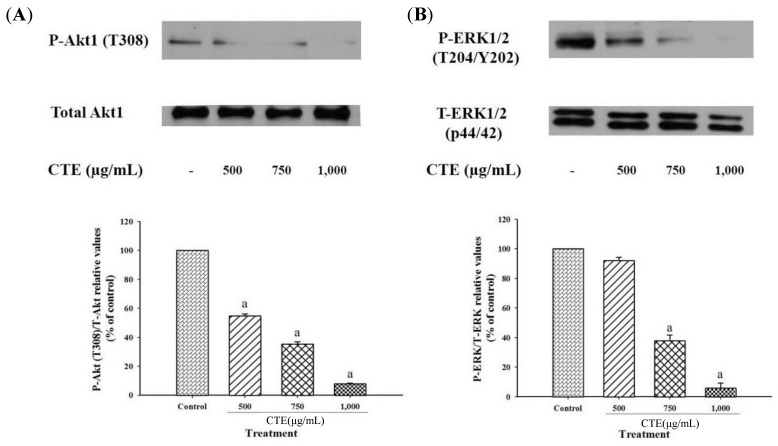
Effects of CTE on Akt (**A**) and ERK (**B**) pathway of 3T3-L1 cells at day 9. The relative values of P (phospho)-Akt1 (T308) to T (total)-Akt1 and P-ERK1/2 (T204/Y202) to T-ERK are shown in % of control. Each value represents the mean ± SEM (*n* = 3). ^a^ *p* < 0.05 compared with the control group.

The results demonstrated the CTE at concentrations of 500, 750 and 1000 µg/mL significantly reduced the phosphorylation level of Akt1 (T308), which ranged from 45.16 ± 1.37% to 91.95 ± 0.31%, as compared to the control (Figure 5A). Furthermore, CTE at concentration of 750 and 1000 µg/mL also significantly suppressed the phosphorylation of ERK1/2 (T202/Y204) about 62.15% and 94.04%, respectively (Figure 5B). However, CTE (500 µg/mL) did not significantly alter the phosphorylation level of ERK1/2.

### 2.5. Effects of CTE on Lipid Accumulation in 3T3-L1 Cells

**Figure 6:**
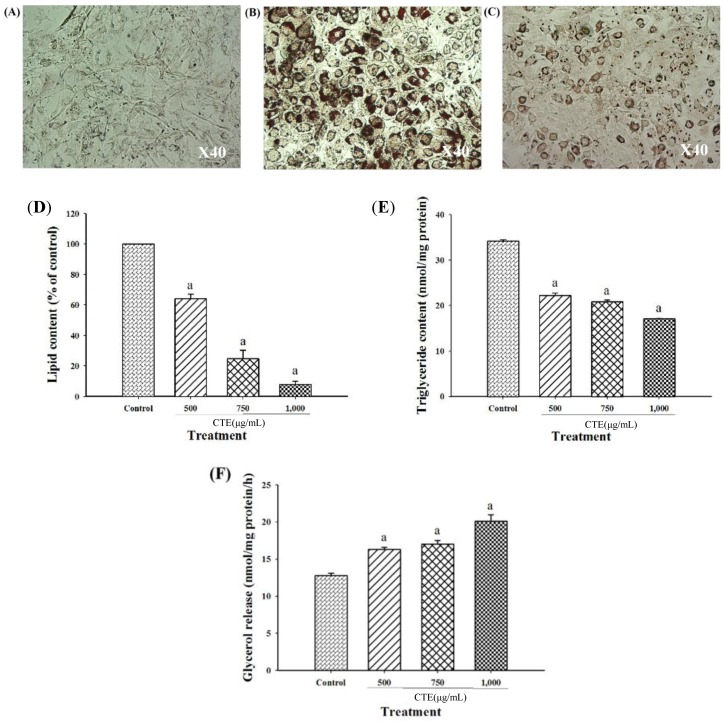
Effects of CTE on lipid accumulation and lipolysis of 3T3-L1 cells at day 9. Undifferentiated cells (**A**), differentiated cells (**B**) and 1000 µg/mL CTE (**C**) were stained by Oil red O (40× magnification). The quantification of lipid content is shown in % of control (**D**). Triglyceride content is shown in nmol/mg protein (**E**). Lipolysis is shown as glycerol release in nmol/mg protein/h (**F**). Each value represents the mean ± SEM (*n* = 3). ^a^ *p* < 0.05 compared with the control group.

After cells were exposed to the inducer including dexamethasone, insulin, and IBMX, 3T3-L1 cells were differentiated to mature adipocytes, as characterized by triglyceride-rich lipid droplet structure histologically. The result demonstrated that CTE (500–1000 µg/mL) significantly decreased Oil-red O staining lipid droplet (26.40 ± 2.14%, 52.86 ± 2.33% and 68.25 ± 2.78%) when compared to control (Figure 6A–D). Furthermore, the accumulation of triglyceride was significantly lowered when the cells was incubated with CTE at the concentration of 500, 750 and 1000 µg/mL (59.05–74.30%), as shown in Figure 6E. The effects of CTE on lipolysis in 3T3-L1 cells is shown in Figure 6F. After 3T3-L1 cells were turned into mature adipocytes, they were exposed to various concentrations of CTE (500–1000 µg/mL) for 1 h. CTE (500–1000 µg/mL) significantly increased lipolysis in a concentration-dependent manner (27.90–57.88%).

### 2.6. Effects of CTE on Adipogenic Transcription Factors and Lipogenic Enzymes

**Figure 7:**
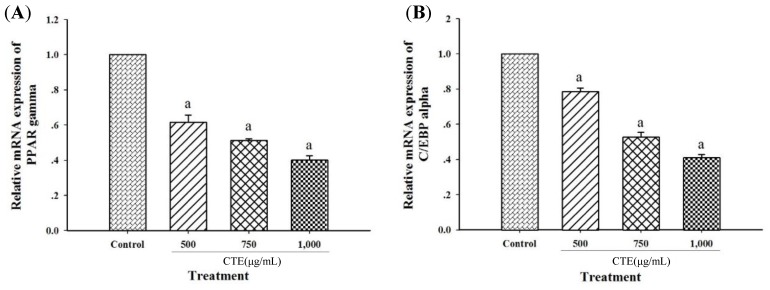
Effects of CTE on mRNA expression of PPARγ (**A**) and C/EBPα (**B**) of 3T3-L1 cells at day 9. The mRNA expression of PPARγ (A) and C/EBPα (B) are shown in relative values to beta-actin. Each value represents the mean ± SEM (*n* = 3). ^a^ *p* < 0.05 compared with the control group.

In respect to Figure 7A,B, CTE (500–1000 µg/mL) significantly suppressed mRNA expression of PPARγ (38.33 ± 3.96%–59.83 ± 2.50%) and C/EBPα (21.49 ± 2.22%–58.94 ± 1.74%).

**Figure 8:**
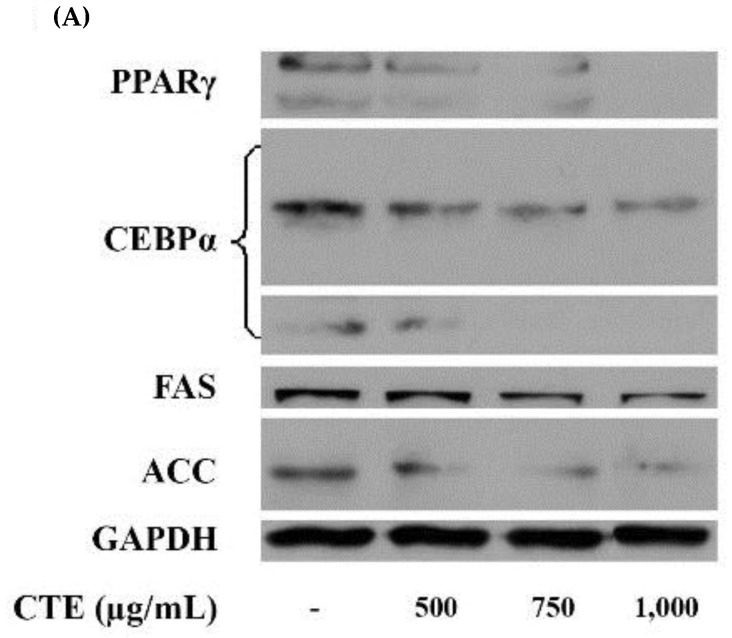

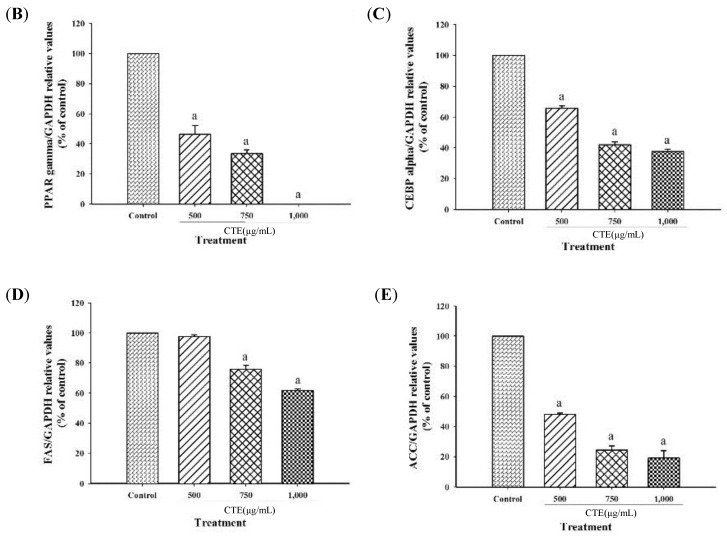
(**A**) Effects of CTE on protein levels of adipogenic transcription factors of 3T3-L1 cells at day 9. The relative values of PPARγ (**B**), C/EBPα (**C**), FAS (**D**), ACC (**E**) to GAPDH are shown in % of control. Each value represents the mean ± SEM (*n* = 3). ^a^ *p* < 0.05 compared with the control group.

Figure 8A demonstrates the protein expression of adipogenic transcription factors and lipogenic enzymes in 3T3-L1 cells. As shown in Figure 8B,C, CTE (500–1000 µg/mL) reduced the protein level of PPARγ (53.50 ± 5.71%–99.99 ± 0.01% and C/EBPα (34.36 ± 1.78%–62.16 ± 1.43%). As illustrated in Figure 8D,E, CTE (750–1000 µg/mL) significantly decreased the level of FAS whereas 500–1000 µg/mL could reduce the protein expression of ACC.

## 3. Discussion

It has been demonstrated that the inhibition of adipogenesis involves the reduction in both number and lipid content of adipocytes [27]. Suppressing differentiation and proliferation, and lipogenesis as well as stimulatory lipolysis are the strategies for prevention and treatment of obesity [17]. It has been reported that medicinal plants and their bioactive compounds possess anti-obesity activity [28]. For example, blueberry peel, *citrus aurantium* and curcumin extracts inhibit adipogenesis through down-regulation of PPARγ and C/EBPα, resulting in decreased cell number and lipid accumulation of adipocytes [29,30]. Recent findings indicated that the blockage of cell cycle through inhibition of Akt1 and ERK1/2 signaling pathway effectively prevents cell proliferation, expression of adipogenic transcription factors, and lipid accumulation in adipocytes [7,14,29,31]. Our current results demonstrated that CTE inhibited the early stage of adipogenesis in 3T3-L1 cells through the suppression of Akt1 and ERK1/2 signaling pathways. Inhibition of this pathway might result in the reduction of hyperplasia in adipocytes [32]. In addition, CTE increased cell cycle arrest by inversion of cell cycle from G2/M to G0/G1 phase. This effect was linked to the suppression of phosphorylated Akt1 at T308 and ERK1/2 at T202/Y204. Many studies have been reported that the phosphorylation of Akt (T308 or ser473) and ERK1/2 (T202/Y204) at the active sites is required for stimulating adipogenesis [13,33,34,35]. For instance, *Coptis chinensis* extract exerts anti-adipogeneic effects via reduction of phosphorylation of ERK1/2 (T202/Y204) and Akt1 (T308) during adipogenesis which was consistent with the current studies [36]. Moreover, kaempferol delayed cell cycle progression by blocking the phosphorylation of Akt [37]. After cells were exposed to the adipogenic inducers including IBMX, insulin and dexamethasone at the late stage, 3T3-L1 cells differentiated to mature adipocytes, resulting in the lipid accumulation. Our findings were consistent with previous studies [37]. In the present study, increased expression of transcription factors including PPARγ and C/EBPα was observed at both mRNA and protein levels. The activation of PPARγ and C/EBPα directly regulates the expression of FAS and ACC leading to conversion of acetyl-CoA to malonyl-CoA and then malonyl-CoA to saturated fatty acids driving formation of triglyceride [38]. Furthermore, the accumulation of triglyceride increases the size of adipocytes [6]. There are several studies reporting that suppressing mRNA or protein expression of FAS markedly prevents the accumulation of triglyceride in adipocytes [29,30,39,40]. For example, cocoa tea water extract inhibits lipogenesis by suppressing expression of lipogenic transcription factors and their target genes such as PPARγ, C/EBPα, FAS, and ACC leading to reduced accumulation of triglyceride in 3T3-L1 cells [41]. Our results showed that CTE dramatically inhibited the late stage of adipogenesis through the inhibition of triglyceride accumulation and expression of adipocyte-associated transcriptional factors and enzymes including PPARγ, C/EBPα, FAS and ACC. Moreover, lipolysis is generally induced by hormone-sensitive lipase (HSL) and consequently released free fatty acid and glycerol and decreased the cell size of adipocytes [42]. It has been reported that black soybean enriched anthocyanin could increase the lipolysis of 3T3-L1 cells, thereby, reduction of cell mass and size [43]. Additionally, polyphenol rich-white tea extract exerts the inhibitory effect on adipogenesis through an increased lipolysis concomitant with suppression of adipogenic markers including SREBP-1c and PPARγ [44]. Suppression of phosphorylated Akt could stimulate HSL in 3T3-L1 cells [45]. The current findings indicated that CTE enhanced lipolysis related to activation of hormone sensitive lipase in adipocytes. The results from this effect cause a decrease in TG accumulation in adipocytes.

Previous studies have demonstrated that CTE contains delphinidin-3,5-glucoside, delphinidin-3-glucoside, malvidin-3-glucoside, kaempferol, quercetin-3-*O*-(2-rhamnosyl) rutinoside, rutin, and six major delphinidin-based ternatins (ternatins A1–A3, B1–B4, C1–C5 and D1–D3) [19,25,26,46]. In the similar pattern, the major bioactive compounds in CTE are on the basis of flavanol glycosides and delphinidin derivatives in this study. It has been clearly shown that several phytochemical compounds exert the inhibitory effect on adipogenesis [27]. For instance, dietary flavonoids including kaempferol, quercetin and myricetin suppressed the adipogenesis by inactivation of Akt1 pathway leading to inhibiting of cell cycle progression, lipid accumulation and transcriptional factor expression in 3T3-L1 cells and human adipose tissue-derived mesenchymal stem cells [36,47]. Rutin could suppress the adipocyte differentiation in 3T3-L1 cells by down-regulating of PPARγ and C/EBPα [48]. As aforementioned, CTE exerts inhibitory effects on adipogenesis by attenuating proliferation of cell, progression of cell cycle (Akt and MAPK), expression of adipogenic transcription factors (PPARγ and C/EBPα) and lipogenic enzymes (FAS and ACC) and enhancing lipolysis. The probable mechanism by which CTE exerts anti-adipogenesis may be associated with the identified phytochemical compounds in the extract. Further studies should be done to prove its anti-obesity effect in animal models. In summary, CTE inhibits cell cycle and adipogenesis at both early and late stages through Akt and MAPK signaling pathway. In late stage, CTE reduces lipid accumulation and the expression of adipogenic transcription factors and enzymes together with enhancement of lipolysis in adipocytes. CTE could be a natural food for prevention of adipogenesis and enhancement of lipolysis.

## 4. Materials and Methods

### 4.1. Chemicals

3T3-L1 preadipocytes were purchased from American Type Culture Collection (ATCC, Manassas, VA, USA). Dulbecco’s modified Eagle’s medium (DMEM) and penicillin-streptomycin were purchased from Mediatech (Herndon, VA, USA). Oil red O solution, isobutylethylxanthine (IBMX), dexamethasone, and insulin were purchased from Sigma-Aldrich (St. Louis, MO, USA). Primary antibodies including phospho (P)-Akt1 (T308), total (T)-Akt1, P-ERK1/2 (T202/Y204), T-ERK1/2, C/EBPα, PPARγ, Fatty acid synthase (FAS), Acetyl-CoA carboxylase (ACC) and GAPDH were purchased from Cell Signaling Technology (Boston, MA, USA).

### 4.2. Plant Materials

Dried flowers of *C. ternatea* were purchased from a local herbal shop in Bangkok, Thailand. The plant was authenticated at the Division of Plant Varieties Protection, Department of Agriculture, Ministry of Agriculture and Cooperatives, Bangkok, Thailand. A specimen was deposited at the institute herbarium under code no: BK066793. The extraction of the plant followed a previously published method [21]. Briefly, the dried plant was extracted with distilled water at 95 °C for 2 h. The sample was then filtered through Whatman 70 mm filter paper. The aqueous solution was dried using a spray dryer SD-100 (Eyela world, Tokyo Rikakikai Co., Ltd., Tokyo, Japan). The spray drying conditions were: inlet temperature (178 °C), outlet temperature (80 °C), blower (0.9 m^3^/min) and atomizing pressure (90 kPa). Total phenolic, flavonoid, and anthocyanin content were 53 ± 0.34 mg gallic acid equivalents/g dried extract, 11.2 ± 0.33 mg catechin equivalents/g dried extract, and 1.46 ± 0.04 mg cyanidin-3-glucoside equivalents/g dried extract, respectively [21].

### 4.3. Sample Preparation for LC-MS/MS

Anthocyanin fractions were obtained using an activated Oasis HLB cartridge (Waters Corp., Milford, MA, USA), according to the modified procedure of previous study with minor modification [49]. Briefly, the dried CTE was resolubilized in distilled water and applied to an activated Oasis HLB cartridge. The cartridge was washed with 0.01% hydrochloric acid in water, followed by ethyl acetate and then 0.01% hydrochloric acid in methanol to elute anthocyanins. Then, the eluent was dried with nitrogen gas and used for LC-MS/MS analysis. The purified CTE was resolubilized with 0.5% formic acid solution to give a final concentration of 1 mg/mL and filtered through a 0.45 µm pore size poly-(tetrafluoroethylene) (PTFE) membrane syringe filter (Corning, New York, NY, USA) prior to injection into the LCMS/MS for identification and quantification of anthocyanins.

### 4.4. Characterization of CTE

The phytochemical compound in the enriched extract were directly analyzed by liquid chromatography and tandem mass spectrometry (LC-MS/MS) according to the conditions previously described by a previous study [25]. LC-MS/MS was performed using a Quadrupole/Time-Of-Flight Mass Spectrometer (QTOF LC-MS/MS; Model-6540 UHD Agilent Technologies, Santa Clara, CA, USA) using a Dual ESI ion source attached to an Agilent 1260 series liquid chromatograph. A 150 × 4.6 mm, particle size 5 μm C18 reversed phase column (VertiSep™ AQS—Vertical Chromatography Co., Ltd., Nonthaburi, Thailand) was used at a flow rate of 0.5 mL/min and 40 min of the total run time. The HPLC gradients were consisted of eluent A; 0.1% formic acid in water and eluent B 0.1% formic acid in acetonitrile. The system was run with the following gradient program: 0 min: 95% of linear gradient until 5% of A in 40 min and post run for 5 min. The mass spectral data were acquired with the following ESI inlet conditions: the scanning mass-to-charge (*m*/*z*) ranging from 100 to 1700 with a scan rate of 4.00 spectra s^−1^, the capillary voltage of 3500 V (positive mode) and the fragmentor of 100 V. The pressure of the nebulizer was set at 30 psi, the drying gas temperature at 350 °C, and the continuous gas flow to 10 L min^−1^.

### 4.5. Culture and Differentiation of 3T3-L1 Cells

3T3-L1 cells were cultured and differentiated as described in the ATCC’s instructions [50]. Briefly, cells were grown in DMEM containing 10% fetal bovine serum, 2 mM l-glutamine, 100 IU/mL penicillin, and 100 µg/mL streptomycin. After cells reached 100% confluence, they were grown in the DMEM supplemented with 10 µg/mL insulin, 0.5 mM IBMX, and 1 µM dexamethasone (differentiation medium) for 5 days. At day 5 until day 9, cells were then grown in DMEM supplemented with only 10 µg/mL insulin (post-differentiation media).

### 4.6. Cell Viability Assay

The cell viability of 3T3-L1 cells was also analyzed using a trypan blue assay as reported in a previous study [51] with minor modifications. 3T3-L1 cells (1 × 10^4^ cells/mL) were incubated with CTE (125–2000 µg/mL) for 1, 3 and 9 day. The trypsinized cell suspension were immediately stained with 0.4% trypan blue for 3 min. The viable cells were counted by automated cell counter (BioRad, Hercules, CA, USA). The viability was expressed as the percentage ratio of the number of unstained cells relative to the total cells counted.

### 4.7. Oil Red O Assay

Oil red O assay was performed according to a previously described method with slight modifications [50]. In brief, 3T3-L1 cells were incubated with CTE (500–1000 µg/mL) until day 9 and differentiated mature adipocytes were then fixed by 10% formaldehyde. After the cells were stained with 60% oil red O solution for 10 min, the cells were washed twice with phosphate buffered saline (PBS). Finally, the cells were incubated with 100% isopropanol at 37 °C for 10 min. The absorbance was measured at 540 nm.

### 4.8. Cell Cycle by Flow Cytometry

Cell cycle analysis was performed following a previously published report with slight modifications [51]. After 3T3-L1 cells were incubated with CTE (125–500 µg/mL) for 24 h, cells were trypsinized and fixed with 50% ethanol at 4 °C for 2 h. Cells were then washed and re-suspended in PBS containing 200 µg/mL RNase A and 50 µg/mL propidium iodide for 30 min at room temperature. DNA content was measured by flow cytometry (FC550, Beckman Coulter, Brea, CA, USA) and analyzed using the Flowjo software. Cells at least 1 × 10^4^ counts were made for each sample.

### 4.9. Western Blot Analysis

Western blot analysis was performed following a previously described method [52]. To determine the protein signaling from proliferation stage such as Akt1 and ERK1/2, the protein was extracted after the incubation with growth media without differentiation cocktail. In addition, the protein extraction of 3T3-L1 cells exposed to differentiation media for 9 days was performed to determine the protein signaling differentiation process including PPARγ, C/EBPα, FAS and ACC. Cells were lysed with ice-cold RIPA buffer (50 mM Tris–HCl pH 8.0, 150 mM NaCl, 1% nonionic detergent (NP40), 0.1% sodium dodecyl sulfate (SDS) and 0.5% sodium deoxycholate) containing protease inhibitor (2 mM PMSF) and phosphatase inhibitor (100 mM NaF, 1 mM Na_3_VO_4_). After centrifugation, the protein content was quantitated by using pierce^®^ BCA protein assay kit Thermo Scientific, Rockford, IL, USA). The lysate was resolved by SDS-PAGE and transferred to nitrocellulose membrane. The membrane was then blocked and probed for specific markers by specific primary antibody. After incubation with horseradish peroxidase-conjugated secondary antibody, membrane-bound target protein was developed and detected using chemiluminescent system (Thermo Scientific). The intensity of each band was quantified by using image J software. Relative expression levels of Akt1 and ERK1/2 were depicted as a ratio of P-Akt1 (T308) to T-Akt1 and P-ERK1/2 (T202/Y204) to T-ERK1/2, respectively. Relative expression levels of PPARγ, C/EBPα, FAS and ACC proteins were shown in ratio of PPARγ, C/EBPα, FAS and ACC to GAPDH, respectively.

### 4.10. Triglyceride Accumulation

Triglyceride content was measured by a colorimetric/fluorometric assay kit (Biovision, Milpitas, CA, USA) according to manufacturer’s instruction. Briefly, 3T3-L1 cells were seeded into 96-well plate with differentiation process with CTE (500–1000 µg/mL) until they became mature adipocytes. Thereafter, lipid droplets were extracted by the extraction buffer and triglyceride was then converted to glycerol and fatty acid by using lipase enzyme. The released glycerol was measured at wavelength of 570 nm.

### 4.11. Lipolysis Assay

Lipolysis assay was performed following the manufacturer’s instructions of a lipolysis colorimetric assay kit (Biovision). Briefly, 3T3-L1 cells were seeded into 96-well plate with differentiation process until they turned into mature adipocytes. The cells were then exposed to the CTE (500–1000 µg/mL) at 37 °C for 1 h. Lipolysis was induced by adding synthetic catecholamine into the cultures that activate β-adrenergic receptor and catalyzes the alteration of ATP to cAMP. The activation of hormone-sensitive lipase hydrolyzed triglyceride to glycerol and fatty acids. Glycerol released from 3T3-L1 cells was measured at wavelength of 570 nm. The result was shown as nmol glycerol/mg protein.

### 4.12. Real-time PCR

After treatment of 3T3-L1 cells with CTE, RNA was extracted from 3T3-L1 cells by using RNeasy Plus Mini Kit (Qiagen, Foster City, CA, USA) and cDNA library was synthesized by using an iScript cDNA synthesis kit (BioRad) according to manufacturer’s suggestions. Reverse transcription was performed with 200 ng of total RNA sample, 1× iScript reaction mix and 1XiScript reverse transcriptase. Quantitative analysis of cDNA was performed using CFX96 Touch^TM^ real time PCR detection system (BioRad) and SsoAdvanced^TM^ universal probes supermix (BioRad) according to the manufacturer’s instructions. Briefly, amplification of the target cDNA was carried out with each 10 µL PCR mixtures containing 1 µL cDNA, 5 µL SSO probes, 0.5 µL primers and 3.5 µL nuclease free water. Target cDNA was amplified by using verified commercially available primer pairs (Mm01205647_g1 for Beta-actin, Mm01331626_m1 for Akt1, Mm00440940_m1 for PPARγ and Mm00514283_s1 for C/EBPα, Thermo Fisher Scientific, Pleasanton, CA, USA). The conditions of PCR reaction were set as follows: it was begun by denaturation cycle at 95 °C for 30 min, followed by 95 °C 10 min and 60 °C for 25 min, respectively. The mRNA expression was normalized with beta-actin following by using 2^−ΔΔCT^ method. Relative gene expression was expressed as fold change in mRNA expression level compared with control.

### 4.13. Statistical Analysis

Data were expressed as mean ± standard error of mean (SEM), n = 3. The statistical significance was evaluated by one-way ANOVA and Tukey’s HSD test (PASW Statistics 18, SPSS Inc., Chicago, IL, USA). *p* < 0.05 was considered to be statistically significant.

## Figures and Tables

**Table 1:** Chromatographic MS and MS/MS data and the chemical structure of identified compounds in CTE.

No.	R_t_ (min)	Compound	Product Ion
**1**	11.098	Preternatin A3	1405.3310 [M + H]^+^, Calcd Mass: 1405.3876, MS/MS: 1329.2817, 1183.2193, 1021.2119, 876.1811, 739.1818, 627.1281, 493.3180, 471.3372
**2**	11.826	Delphinidin derivatives	788.4017 [M + H]^+^, Calcd Mass: 789.2084, MS/MS: 801.3825 [M + Na]^+^, [Quercetin 3-glucoside+dihexose]
**3**	12.277	Ternatin B2	1637.3591 [M + H]^+^, Calcd Mass: 1637.4248, MS/MS: 1389.3186, 757.1914, 611.1385, 465.0866, 303.0396
**4**	12.797	Ternatin D2	1475.3163 [M + H]^+^, Calcd Mass: 1475.3720, MS/MS: 741.1967, 595.1441, 449.0916, 287.0456
**5**	13.040	Quercetin-3-rutinoside (rutin)	611.1384 [M + H]^+^, Calcd Mass: 610.1534, MS/MS: 465.0860, 303.0399
**6**	13.109	Ternatin D1	1697.3959 [M + H − Malonyl]^+^, Calcd Mass: 1697.4612, MS/MS: 1389.3165, 1243.2461, 611.1379, 465.0855, 303.0396
**7**	13.664	Kaemferol-3-*O*-(2-rhamnosyl)rutinoside	741.1969 [M + H]^+^, Calcd Mass: 740.2164, MS/MS: 595.1443, 449.0924, 287.0457
**8**	14.080	Delphinidin-3-glucoside (myrtillin)	465.0856 [M + H]^+^, Calcd Mass: 465.1028, MS/MS: 487.0665 [M + Na]^+^, 303.0390
**9**	14.218	Kaemferol-3-*O*-rutinoside	595.1437 [M + H]^+^, Calcd Mass: 594.1585, MS/MS: 588.3886, 566.4074, 449.0917, 287.0454
**10**	14.738	Delphinidin-3-*O*-(6-*O*-*p*-coumaryl)glucoside-pyruvic acid	679.4869 [M + H]^+^, Calcd Mass: 679.1294, MS/MS: 701.4680 [M + Na]^+^, 595.1422, 340.2488
**11**	15.4311	(+)-Catechin 7-*O*-β-glucoside	453.3275 [M + H]^+^, Calcd Mass: 452.1319, MS/MS: 927.6276 [2M + Na]^+^, 905.6462 [2M + H]^+^, 814.5468
**12**	15.639	Syringetin-3-*O*-glucoside	509.8675 [M + H]^+^, Calcd Mass: 508.1217, MS/MS: 1040.7075 [2M + Na]^+^
**13**	16.402	Quercetin triglycoside	759.3755 [M + H]^+^, Calcd Mass: 758.1906, MS/MS: 781.3571 [M + Na]^+^

## Abbreviations

ACC: Acetyl-CoA carboxylase; C/EBPα: CCAAT/enhancer-binding protein alpha; CTE: *Clitoria ternatea* extract; ERK1/2: Extracellular signal–regulated kinases 1 and 2; FAS: Fatty acid synthase; IBMX: 3-isobutyl-1-methylxanthine; PPARγ: Peroxisome proliferator-activated receptor gamma.
